# Could the Arginine-Fluoride Association Have a Real Impact on Caries Prevention?

**DOI:** 10.7759/cureus.72153

**Published:** 2024-10-22

**Authors:** Sara El Harram, Tarik Sqalli

**Affiliations:** 1 Conservative Dentistry and Endodontics, Faculty of Medicine, Pharmacy and Dentistry, Sidi Mohamed Ben Abdellah University, Fes, MAR; 2 Laboratory of Epidemiology and Research in Health Sciences, Faculty of Medicine, Pharmacy and Dentistry, Sidi Mohamed Ben Abdellah University, Fes, MAR

**Keywords:** arginine, association, biological approach, caries prevention, fluoride, toothpaste, varnish

## Abstract

In the context of preventive therapy, fluoride has allowed the reduction of the incidence and prevalence of caries. The establishment of the protective role of arginine in the neutralization of plaque acids has led to the development of new arginine-fluorine associations to potentiate this preventive aspect. Studies have reported that this association could provide superior efficacy compared to fluoride alone. A synergistic effect that could be potential and thus represent a promising ecological approach for caries prevention. However, confirmation of the effective efficacy of the arginine-fluoride association requires additional clinical studies to be carried out before establishing recommendations.

## Introduction and background

Caries disease is the result of a multifactorial process that can be arrested and reversed during the earliest stages. Thus, a preventive approach remains the most effective way to reduce the prevalence of this disease [1-3]. Fluoride plays a key role in preventing and controlling the progression of carious lesions and has a potential impact in decreasing caries prevalence [3,4]. While the role of fluoride in remineralization and demineralization dynamics is well established, its effect on cariogenic biofilms is limited and unsustainable [5]. Given the effective action of fluoride, it would be wise for any new caries management strategy to complement its effects by targeting biofilm pathogenicity [5]. Many agents, ranging from antimicrobials to microbiome modifiers, have been advocated to support the action of fluoride (F) in combating biofilm dysbiosis [6-8]. One promising approach involves combining fluoride with arginine, a naturally occurring prebiotic found in saliva identified as a critical factor that increases pH [9]. This synergistic combination has shown potential in modulating cariogenic biofilms, enhancing fluoride uptake, and promoting remineralization. The arginine-fluoride association has been proposed in various topical vehicles, both self-administered and professional. Some of these products are already in the market, while others remain in the pre-clinical stages of investigation. Some in vitro and clinical studies have been conducted to evaluate this association, but the literature is still seeking solid evidence to provide relevant conclusions.

This article examines the dynamic nature of the caries process, highlighting the roles of fluoride and arginine, as well as the potential benefits of their combined application across various delivery systems, including toothpaste and varnishes.

## Review

The dynamic nature of the caries process

The formation of an initial caries lesion involves the interaction over time between acidogenic bacteria encased in a biofilm, fermentable carbohydrates, the tooth surface, and the oral environment. This complex and dynamic process occurs at the interface between the tooth surface and the bacterial biofilm [3]. A high and continuous consumption of fermentable sugars leads to acid production by acidogenic bacteria, causing significant changes in the balance of the biofilm [1,2,10,11]. These acidogenic bacteria are also aciduric, enabling them to survive in the acidic conditions they create at the tooth-biofilm interface, unlike other bacterial species that could not survive in this environment. Consequently, the biofilm previously compatible with dental health becomes cariogenic [2,6,10,12]. If acid production continues over time, the pH will drop further until it reaches the critical threshold of 5.5, leading to the dissolution of calcium and phosphate ions and the demineralization of calcified dental tissues. As long as the pH remains below this critical threshold, demineralization continues and the initial lesion of the enamel sets in [1,13]. Saliva has an important protective role through its ability, among other things, to buffer bacterial acids. It also intervenes through its action of clearing fermentable sugars [5,14]. When the pH increases, the calcium and phosphate ions present in saliva and the bacterial biofilm will be able to return to the enamel surface and participate in its remineralization [14]. However, when salivary flow is compromised or sugar consumption remains high, the balance between demineralization and remineralization can shift unfavorably, allowing carious lesions to progress [1,2].

The main way to act on the caries process during the early stages is the effective and regular elimination of the biofilm. However, this effective control of bacterial biofilm is difficult to achieve, particularly due to lack of motivation and dexterity, the adoption of a cariogenic or unbalanced diet, and hence the addition of anti-cariogenic chemical agents as part of preventive therapy [15]. Among these agents, fluoride is considered the most used in the prevention and treatment of carious disease.

Role of fluoride in the prevention of carious disease

One of the primary objectives in preventing or treating initial carious lesions is to strengthen calcified dental tissue, making it less vulnerable to acid attacks [3,16]. This objective has prompted the systemic introduction of fluorides through sources such as drinking water, food, and supplements, aimed at enhancing enamel resistance during its formation. However, the systemic use of fluoride is currently being reevaluated due to potential toxicity concerns [3]. Recent research highlights that fluoride predominantly exerts its cariostatic effects at the mineral surface and oral fluid interface, where it can effectively intervene in the carious process [3,16]. Consequently, topical fluoride applications, such as those found in toothpaste, mouth rinses, and floss, are recognized as a major factor in decreasing caries prevalence when integrated into daily oral hygiene practices [17].

Topically applied fluoride provides a dual mechanism of action: it inhibits demineralization and enhances remineralization. Following an acid attack, fluoride ions are adsorbed onto the hydroxyapatite crystal surfaces, forming a protective coating that inhibits dissolution. Saliva serves to buffer the acidic environment and elevate the pH. The natural remineralization process is augmented by saliva, which is supersaturated with calcium and phosphate ions. The presence of fluoride enhances this process, as it preferentially attracts these ions, leading to the formation of fluorapatite crystals, which are more resistant to dissolution than hydroxyapatite, and only soluble below a pH of 4.5 [4,13,16].

Furthermore, fluoride possesses inherent antibacterial properties. At acidic pH levels, fluoride ions can penetrate bacterial cells in the form of hydrofluoric acid (HF), which subsequently dissociates into H+ and F- ions. The H+ ions lower the intracellular pH, while the F- ions inhibit essential metabolic pathways by targeting enolase, an enzyme critical for glycolysis. Additionally, F- ions disrupt H+/ATPase pump function, preventing the restoration of intracellular pH [9,13,16]. Despite extensive data in the literature on this subject, fluoride has not demonstrated a sustained impact on cariogenic biofilms, even at high concentrations. This is because the preventative effect of fluoride on dental caries reaches a saturation point, beyond which additional fluoride concentration does not significantly enhance its efficacy [18]. Moreover, long-term use of fluoride has demonstrated resistance to cariogenic pathogens such as *Streptococcus mutans* [19]. Therefore, it is essential to combine the benefits of fluoride with strategies aimed at modulating the cariogenic biofilm and maintaining homeostasis [20].

Role of arginine in the prevention, arrest, and reversal of carious disease

Kleinberg's (1999) research on the action of saliva in modulating pH has highlighted the role of arginine in raising pH and maintaining supragingival ecological homeostasis, as well as preventing biofilm dysbiosis [9,21-23]. Unlike cariogenic bacteria that can survive in acidic environments due to their acidogenic and aciduric properties, certain bacterial species, such as *Streptococcus sanguinis* and *Streptococcus gordonii*, present in the biofilm have difficulty surviving at the biofilm-tooth interface because they lack this tolerance to acidity [5]. However, they process an adaptive mechanism known as the arginine deiminase pathway, which breaks down arginine, a semi-essential amino acid naturally present in saliva, to produce energy, ammonia, and carbon dioxide. Ammonia neutralizes acids and restores the pH of the biofilm, creating a less favorable environment for cariogenic bacteria [5,14,24]. Thus, this prebiotic selectively inhibits the growth of cariogenic pathogens such as *Streptococcus mutans* while promoting the growth of arginolytic commensals (*Streptococcus sanguinis*, *parasanguinis*, *gordonii*…) [25-27]. Given its contribution to pH homeostasis in dental biofilms, Arginine could serve as an important agent for maintaining healthy oral biofilms and thus preventing dental caries [28].

Role of the arginine-fluorine association in the prevention, arrest, and reversal of carious disease

The aim of this approach is to adopt an ecological strategy for managing carious disease by exploiting the potential synergistic effect of the combined application of fluoride and arginine application [9]. This combination would allow for modulation of the biofilm, an increase in pH at the interface, and enhanced fluoride absorption by dental tissues and bacterial plaque, thereby optimizing the potential for remineralization. Arginine may promote fluoride uptake in the enamel of demineralized lesions [29,30], due to its terminal guanidinium group, which attracts electronegative elements, particularly fluoride, leading to the formation of an Arg-F complex. This complex serves as a stable and bioavailable source of fluoride [31-33]. The interaction between arginine and fluoride operates on the principle of organic-inorganic interactions, similar to the mineralization process in natural tissues (biomimetic approaches). Arginine plays a role similar to that of the organic matrix in mineralization [34]. Organic molecules, such as arginine, possess positive charges either on their surface or embedded within structural folds [35]. These molecules act as nucleation centers for mineralization through strong electrostatic interactions, facilitating the precipitation of inorganic minerals. This ecological strategy has led to the incorporation of arginine-fluoride in self-administered vehicles, as well as in vehicles for professional application.

Self-Administered Vehicles: Arginine-Fluoride-Based Toothpaste

The first proposed product integrating this combination is toothpaste. However, only a small number of commercial products are currently available. These products have been examined and compared in a limited number of in vitro and clinical studies, raising questions about the level of evidence needed to draw clear conclusions and recommendations on the synergistic effect of arginine and fluoride in the prevention, arrest, or reversal of caries. While the studies conducted recognize the anti-caries effect of this combination, the interpretation of the results from comparisons with other toothpaste with different formulations must be approached with caution. This is due to the potential risks of bias related to conflicts of interest or commercial contributions in the studies.

Two concentrations of arginine have been proposed in combination with 1450 ppm of fluoride. The 1.5% arginine is the most retained for anti-caries purposes, and 8% arginine is intended for use against dental hypersensitivity. The 8% concentration has also been evaluated for its anti-caries effect. In studies examining the antibacterial effect of the arginine-fluorine association, in vitro studies have shown that toothpastes based on this combination (1.5% arginine-1450 ppm F- and 8% arginine-1450 ppm F-) have a better modulating effect on the biofilm compared to toothpaste with 1450 ppm fluoride alone, in both caries-active and caries-free sites. These toothpastes were associated with a higher proportion of arginine deiminase system-positive strains (*S. sanguinis* and *S. gordonii*), increased pH [26], and lower concentrations of inorganic and insoluble extracellular polysaccharides (IEPS) in the biofilm [36].

Long-term use of arginine-fluoride dentifrice toothpaste (1.5% arginine-1450 ppm F⁻) has demonstrated a significant shift in the bacterial composition and functional profiles of human dental plaque towards a healthier microbial community, as observed in clinical trials [37]. Additionally, the use of toothpaste with 8% arginine and 1450 ppm F⁻ has been shown to effectively decrease the number of *Streptococcus mutans* in dental plaque around orthodontic brackets when compared to traditional fluoride toothpaste (1450 ppm F⁻) [38]. This formulation has also significantly reduced lactic acid production in in-situ plaques to low levels without impacting metabolic activity, the live-to-dead bacteria ratio, or the overall biofilm biomass [39]. Furthermore, this same concentration has exhibited a favorable modulating effect on the oral microbiota of individuals with active caries, as evidenced by cohort studies [40].

The combination of 1.5% arginine and 1450 ppm fluoride has shown potential for enhancing fluoride absorption and reducing enamel solubility at the enamel surface and within the plaque, comparable to toothpaste containing 1450 ppm fluoride (sodium monofluorophosphate) and even 2500 ppm fluoride (sodium monofluorophosphate). However, its efficacy remains significantly lower than that of combined formulations (1500 ppm sodium fluoride (NaF) + 1000 ppm sodium monofluorophosphate) [41]. Moreover, this arginine-fluoride-based toothpaste has also demonstrated resistance to demineralization, showing similar effectiveness to various other toothpastes studied [41]. While arginine appears to enhance the effect of fluoride at ordinary concentrations, toothpastes with higher fluoride content still hold a clear advantage. A randomized clinical trial suggested that a toothpaste with 1.5% arginine and 1450 ppm fluoride is significantly more effective in stopping and reversing initial root lesions compared to a fluoride toothpaste with 1450 ppm NaF [42]. However, this efficacy remains lower than that of toothpaste with a high fluoride content of 5000 ppm [43]. Similarly, in vitro studies have shown that toothpaste with 1.5% arginine-1450 ppm fluoride continues to demonstrate a remineralizing effect, though still lower than toothpaste containing 1450 ppm fluoride associated with other agents, such as hydroxyapatite and various enzymes designed to potentiate fluoride's effect [44].

Clinically, the long-term effects of using these toothpastes lack substantial evidence due to the limited number of studies and the high risk of bias associated with them. Randomized double-blind clinical trials have reported a statistically significant reduction in CAOD (caries, absences, and obturations on permanent teeth) and CAOF (caries, absences, and obturations on deciduous teeth) indices after two years of using toothpaste containing 1.5% arginine and 1450 ppm fluoride compared to toothpaste containing 1450 ppm fluoride alone. These studies concluded a positive anti-caries effect of the arginine-fluoride toothpaste compared to the control [45-47]. Additionally, other double-blind randomized clinical trials also reported a statistically significant reduction in the volume of initial lesions after six months of using the arginine (1.5%)-fluoride (1450 ppm) toothpaste, compared to fluoride-only toothpaste [48,49]. However, further validation of these effects necessitates longer-duration studies with larger sample sizes.

The 8% arginine-1450 ppm toothpaste was compared to other non-fluoridated toothpaste containing bioactive elements, such as casein phosphopeptides-amorphous calcium phosphate (CPP-ACP) and glass particles containing calcium phosphate (NovaMin technology). These products can supersaturate the dental environment with high concentrations of calcium and phosphate, allowing these chemical elements to penetrate the tooth and precipitate through the normal process of osmosis. Toothpaste containing arginine-fluoride and NovaMin technology showed a significantly higher surface hardness and lower surface roughness compared to those treated with CPP-ACP [50]. This association showed comparable efficacy to toothpaste containing fluoride with other technologies: REFIX technology, NR-5 technology, and NovaMin technology [51]. However, the results regarding the remineralization potential of this concentration of arginine-fluoride remain inconclusive, and its advantages are unclear. This uncertainty has prompted a study to determine the optimal arginine concentration for remineralization in the arginine-fluoride association. The study compared 2%, 4%, and 8% L-arginine in NaF toothpaste, suggesting that 2% arginine in 1100 ppm NaF toothpaste significantly increased enamel remineralization compared to 1100 NaF toothpaste alone. Conversely, 4% and 8% arginine in NaF toothpaste were less effective in enhancing enamel remineralization. This reduced efficacy at higher concentrations may result from the overabundance of L-arginine monohydrochloride in the 8% Arg-NaF toothpaste, potentially hindering the interaction between L-arginine monohydrochloride and sodium fluoride, thereby limiting the availability of free fluoride ions compared to the 2% Arg-NaF formulation [29]. Thus, the various technologies proposed to enhance the remineralizing effect of anti-caries toothpaste should be compared to Arg-F toothpaste containing different concentrations of arginine to draw more relevant conclusions on the optimal concentration that provides the most effective anti-caries benefit.

Professional Application of the Arginine-Fluorine Association

The incorporation of arginine and fluoride in professional application vehicles, particularly varnishes, has gained attention in dental research. While only one study has incorporated 4% arginine into a commercially available glass ionomer, noting an improvement in antimicrobial properties, it did not stimulate further investigation in this area [52]. Fluoride varnish is recommended for high-caries-risk patients because it adheres to the tooth surface, thus prolonging the contact time between fluoride and the tooth surface [53]. This property can be exploited in professional arginine administration through its inclusion in a fluoride varnish, which could provide a synergistic anti-caries effect. This combination would target biofilm control through arginine and enamel remineralization via fluoride [54]. This approach would likely enhance the effect of fluoride without increasing its already high concentration in the varnish. One of the first studies exploring a customized 3% arginine varnish highlighted its relevance in plaque control and suggested its potential as an adjunct to oral hygiene measures for high-risk patients [55]. However, targeted professional administration requires additional evaluation steps before its standardization for clinical use [56].

Few studies have investigated the effect of incorporating arginine at different concentrations into fluoride varnish. Research indicates that adding 2% L-arginine to 5% NaF varnish improves its physical properties, limiting flow and increasing retention on tooth surfaces. This concentration produces a stable matrix with a slow and sustained release of the Arg-Ca-F2 complex in the presence of acidic microenvironments and a release of bioavailable F ions over a prolonged period [31,54,57,58]. The fluoride uptake, calcium/phosphate (Ca/P) ratio, arginine uptake, mineral gain, and percentage remineralization for 2% arginine and 5% NaF varnish were significantly higher than those for 5% NaF varnish alone [54,57]. However, with higher concentrations of arginine (≥ 4%), as seen with arginine-fluoride toothpastes, the fluoride release dynamics are accelerated, leading to a rapid depletion of fluoride reserves in the matrix and, therefore, limited availability of fluoride in the biofilm and the surface of adjacent hard tissues. Moreover, the presence of a large amount of free fluoride does not improve the remineralization effect compared to 2% arginine [31,57]. Furthermore, an 8% arginine concentration in a 5% NaF varnish showed significantly higher cytotoxicity to gingival fibroblasts than the 5% NaF varnish, likely due to uncontrolled fluoride release from the matrix of the 8% Arg-NaF varnish [54]. Further studies are needed to explore whether higher concentrations of L-arginine (8%) could lead to biofilm issues related to alkalinization and potentially promote the growth of periodontal pathogens, including *Porphyromonas gingivalis* [24].

The addition of 2% and 4% arginine to a 5% NaF varnish improved its potential to modulate cariogenic biofilm by reducing biofilm thickness, increasing the proportion of dead cariogenic bacteria, and decreasing carbohydrate content [32,33]. Given that both 2% and 4% arginine in 5% NaF exhibited similar effects on biofilm pathogenicity modulation, the 2% Arg-NaF varnish emerges as a promising concentration for incorporation into a 5% NaF varnish for professional applications. This concentration significantly enhanced enamel fluoride uptake and the remineralization potential of conventional 5% NaF varnish [31,32,54] by providing a stable matrix that allows for sustained fluoride release over an extended period [57,59].

The professional application of fluoride varnish incorporated with arginine remains exploratory, especially since studies have primarily tested this arginine-fluoride combination in Duraphat® varnish [58]. Thus, evaluating this association in other varnishes with varying fluoride concentrations is necessary to provide more evidence regarding the effectiveness of this approach in caries prevention. The incorporation of 10% arginine in a 5% NaF varnish with CPP-ACP and a 10% nano-hydroxyapatite varnish showed a significant increase in finishing values and mineral gain at the enamel level, suggesting a synergistic effect of arginine with fluorides and nano-hydroxyapatite, thereby enhancing their role in remineralization [60]. A comparison of the effect of incorporating 2% arginine into varnishes with different fluoride concentrations (Duraphat® 5% NaF, Fluor Protector® 0.9% SiH2F2, Fluor Protector S® 1.5% NH4F, Fluor Protector S® Fluorimax™ 2.5% NaF) showed that this addition was accompanied by an increase in the remineralization potential of the enamel, likely due to the increased fluoride release potential, mainly related to the arginine-fluoride interaction in the varnish matrix/suspension [20,58]. However, these results necessitate a minimum acceptable fluoride concentration to achieve better precipitation of calcium and phosphates [30,58]. The study also reported no difference in the percentage of remineralization between Duraphat® (5% NaF) and 2% Arg + Fluorimax™ (2.5% NaF). This result suggests that remineralization can be enhanced twofold in the presence of arginine at appropriate concentrations. However, these interpretations should be approached with caution, due to the analysis of the ingredients in Fluorimax™, which revealed nano-hydroxyapatite that could have a synergistic effect with arginine, significantly enhancing the mineralization potential of the varnish [60]. Therefore, the observed dual remineralization effect can be attributed to both NaF and nano-hydroxyapatite [20,58].

## Conclusions

In conclusion, while the role of arginine, naturally present in saliva, is acknowledged, current in vitro and clinical data are insufficient to provide definitive recommendations on its potential for caries prevention when combined with fluoride agents. Studies, including systematic reviews and meta-analyses, have shown an anti-caries effect in arginine-fluoride toothpaste, but their effectiveness compared to other formulations remains controversial due to concerns about bias and conflicts of interest. Among different concentrations tested, 2% arginine appears to offer the best enhancement to fluoride's anti-caries properties. However, for arginine-fluoride varnishes, most research has been conducted in vitro, and the lack of clinical data makes it challenging to draw firm conclusions. A concentration of 2% arginine in fluoride varnishes seems promising, though it requires a sufficient level of fluoride to maximize its anti-caries potential.
